# Insight into the Micro Evolution of Backfill Paste Prepared with Modified Gangue as Supplementary Cementitious Material: Dissolution and Hydration Mechanisms

**DOI:** 10.3390/ma16196609

**Published:** 2023-10-09

**Authors:** Binbin Huo, Jixiong Zhang, Meng Li, Qiang Guo

**Affiliations:** 1 School of Mines, China University of Mining and Technology, Xuzhou 221116, China; huobinbin@cumt.edu.cn (B.H.); stu-gq@cumt.edu.cn (Q.G.); 2 State Key Laboratory of Coal Resources and Safe Mining, China University of Mining and Technology, Xuzhou 221116, China; limeng77521@126.com

**Keywords:** gangue, backfill, cementitious material, microstructure, hydration

## Abstract

Gangue-based backfill cementitious materials (BCM) are widely applied due to their low CO_2_ footprint, while the application is restricted by gangue’s low reactivity. In this study, dry chemical modification was developed to modify the gangue, and multiple characterized approaches were used to characterize the dissolution property, mineral composition, and particle size distribution of modified gangue (MCG), as well as the compressive strength and microstructure of BCM. The findings show that the residue weight of MCG stabilized at 2 wt.% of formic acid, and the modification reduces the kaolinite and calcite, resulting in smaller particles. Additionally, the three days compressive strength of the BCM made with MCG was improved from 0.3 MPa to 0.6 MPa. Attributed to the increased reactivity of MCG, it was found that the dissolution weight increased by 2.13%. This study offers a novel method for activating gangue and a new kind of MCG-prepared BCM, which makes a significant contribution towards achieving the UN Sustainable Development Goals.

## 1. Introduction

Low carbon cementitious materials (LCMs) are becoming more and more popular in mine backfills [1,2], bridges [3], dams [4], and buildings [5] due to their low cement and high solid waste content, which makes the materials more affordable and have a lower CO_2_ footprint. This is due to the significant environmental impact associated with coal mining and cement manufacturing. The use of LCM is both topical and pressing given the compelling need for sustainable mining [6], cement [7], and environment [8] for partly realizing the UN Sustainable Development Goals (SDGs) by 2030.

However, this trend in low cement unavoidably has effects such as reduced compressive strength and poorer durability, as well as hydration and microstructure, which can be quite different from standard Portland cement binders [9,10]. Although significant efforts have been made to enhance the LCM properties through the addition of activators, increase in cement, and decrease in the water to binder ratio, some studies [11,12] on LCM prepared by an increase in cement or a decrease in the water to binder ratio reported an increase in manufacturing costs and a decline in workability. It has been demonstrated that high activity solid wastes aid in a better pozzolanic reaction in cementitious materials, which can improve the mechanical performance of LCM by optimizing the interface transition zone, resulting in the effective fabrication of LCM with satisfied qualities [13,14,15].

Gangue is a by-product of the coal industry, and its annual emission has reached 600 Mt due to China’s rising energy consumption. Efficient disposal has been a serious issue due to the huge accumulation of gangue. Gangue aggregate or powder is used to make construction materials [16,17,18]. According to the reports [19,20], activated gangue powder can be used as supplementary cementitious material (SCM) to partially replace cement in order to improve the microstructure of cementitious materials. However, un-pozzolanic kaolinite, quartz, and muscovite make up the majority of the gangue. The structure of gangue is stable and the reactivity is low. As a result, the insufficient compressive strength of gangue based cementitious backfill pastes is unable to control the surface subsidence [21,22]. Physical and chemical modifications, such as mechanical grinding, thermal activation, and alkali activation increase in pozzolanic mineral content and mineral dissolving rate have been used to overcome the weakness of gangue activity [23,24]. Of these activating approaches, Guo et al. [25] discovered that thermal calcination was an incredibly effective approach to improve the gangue reactivity because kaolinite transforms to metakaolin between 550 and 950 °C. Záleská et al. [26] evaluated the functional parameters of blended cement pastes containing 10 wt.%, 15 wt.%, and 20 wt.% of thermally treated mining gangue, and found that thermal activated gangue could be safely used to replace cement up to 20%. Based on the calcination, Ma et al. [27] found that adding additional alkali activators, such as NaOH and Na_2_SiO_3_, would encourage the gels to develop and produce high strength geopolymer. Alkali activation and calcination, on the other hand, are not appropriate for widespread industrialization because of their high energy requirements and challenging processing methods. Compared to these two methods, grinding is more appropriate for usage in industry due to its greater operability and lower energy consumption. Moussadik et al. [28] reported that preactivated gangue by method of grinding without calcination could produce amorphous geopolymer gel based on silica-alumina (N-A-S-H gels), as described in Equation (1). These gels fill the pores in matrix and increase the strength of the samples. While Zhou et al. [29] reported that further grinding demonstrates difficulty in deeply activating the gangue at the nano- or molecular-scale, due to mechanical activation technology, cannot reach that scale. According to the findings described above, it is vital to create new approaches for activating gangue with little carbon impact.

Na^+^ + AlO_2_^−^ + SiO_4_^2−^ + OH^−^ → N-A-S-H (1)

Gangue activity improvement is limited by single grinding. Surface modification with adjustors is helpful for the improvement of the gangue property. Wu et al. [16] used slurry to modify the gangue aggregate surface and improved the permeability and connected porosity of gangue concrete. Zhang et al. [30] used phosphotungstate to modify gangue and enhanced the adsorption properties of polyoxometalates/coal gangue composite. Sun et al. [31] prepared a novel composite adsorbent by loading layered double hydroxide on the inner pore wall of a coal gangue “ball–window” porous monolith as the skeleton, and the composites show excellent removal efficiency and recyclability for trace emulsified water from contaminated oil. Guo et al. [32] used water-reducing agents to modify and improve the fluidity, stability, and mechanical properties of gangue based backfill pastes. Recently, a dry chemical modification technique was proposed by Huo et al. [33] to improve the reactivity and soundness of a kind of solid waste, such as steel slag powder. Formic acid, acetic acid, and phosphoric acid were discovered to react with harmful free-CaO in steel slag powder during the modification process and etch with various minerals to produce corresponding salts and cause different minerals to separate from each other. This procedure improves the steel slag powder’s ability to interact with liquid, hastening the process of hydration or dissolution in general. Modification is a potential activating method that has been widely used in alloys [34], biological materials [35], heavy metal adsorption materials [36,37], and civil engineering materials [38]. The chemical modification technique can be combined with the grinding approach, but its use on gangue is still unclear.

The research is motivated by the SDGs of the desire to develop gangue-based LCM with a low CO_2_ footprint. The objectives of this study are to investigate the viability of using a dry chemical modified approach to activate gangue and to increase the mechanical compressive strength of backfill cementitious material (BCM) made from gangue that has undergone dry chemical modification. The gangue was pretreated using a new dry chemical modified method. It was projected that etched gangue treated with various amounts of formic acid would dissolve in alkali. Then, the MCG’s mineral content, shape, and particle size distribution were characterized. The microstructure and compressive strength of the pastes prepared with MCG were also examined. In addition to producing new gangue-based mine functional materials, this will reveal the activated and hydrated mechanisms toughened in dry chemical modified gangue and promote the realization of the goals of SDGs.

## 2. Materials and Methods

### 2.1. Flow Chart of the Research

**Figure 1:**
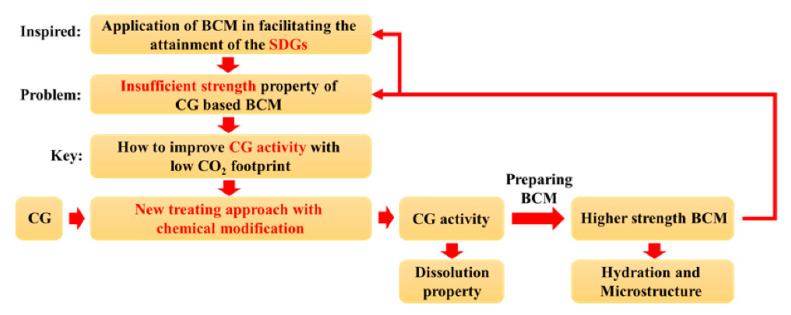
Flow chart of the research.

Figure 1 shows a schematic flow chart of the research and how to approach the SDGs.

### 2.2. Materials

**Figure 2:**
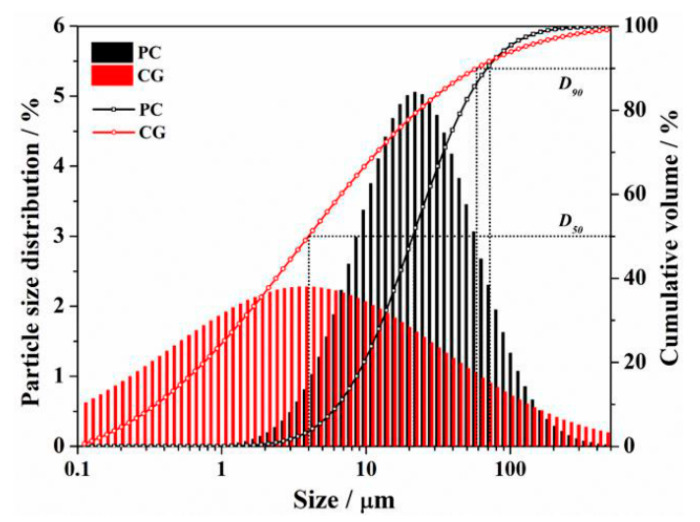
Particle size distribution of PC and CG.

**Figure 3:**
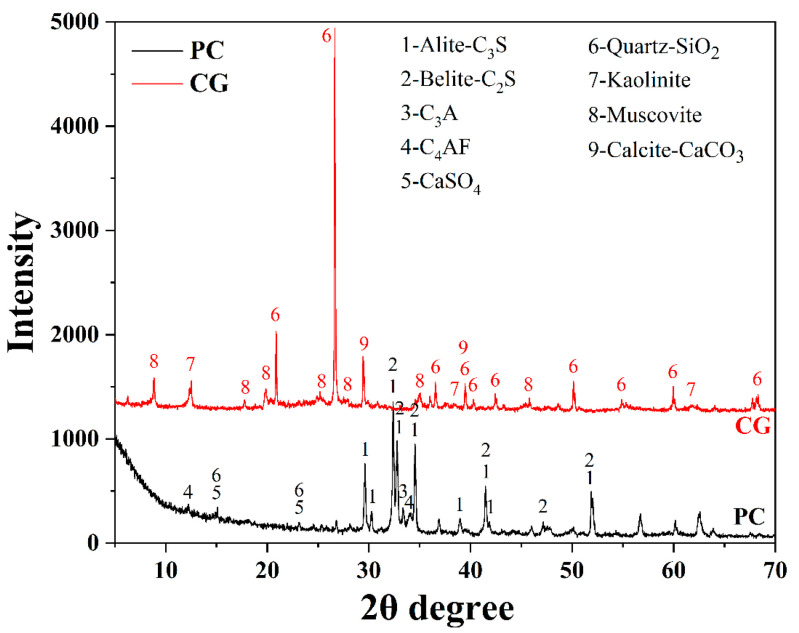
XRD patterns of PC and CG.

The raw binder materials employed in this experiment were PI 42.5 cement (PC) and coal gangue (CG), which were provided by China United Cement Group Co., Ltd. (Nanjing, China) and Shandong Xinjulong Coal Mine Co., Ltd. (Heze, China), respectively. The particle size distributions of PC and CG measured by Laser Particle Size Analyzer are presented in Figure 2. The data shows that the D_50_ of the PC and CG were 11.3 μm and 4.1 μm, respectively, and that of the D_90_ were 73.1 μm and 62.4 μm, respectively. X-ray fluorescence and quantitative X-ray diffraction studies were used to determine the chemical and mineral compositions of PC and CG, respectively, as shown in Figure 3 and Table 1. We also utilized formic acid (FC) as the etchant, whose concentration was 88.0 wt.%.

### 2.3. Sample Preparation

#### 2.3.1. Chemical Modification

**Figure 4:**
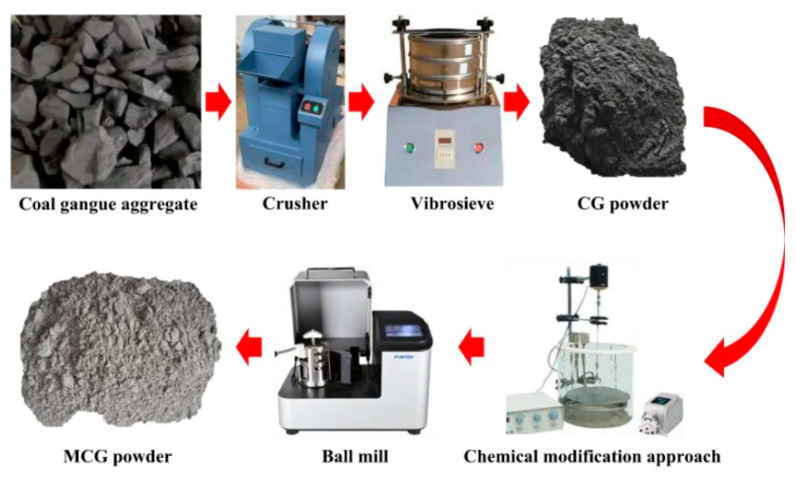
Flow chart of the chemical modification process.

Figure 4 depicts a schematic flow chart of the chemical modification process. The CG aggregate was firstly crushed to produce CG powder and sieved with a particle size lower than 200 mesh. The CG powder was then mixed and subjected to chemical modification equipment using FC. Finally, the modified CG (MCG) was achieved after the CG was next ground for 30 min.

#### 2.3.2. Pastes Preparation

The sample pastes were made in accordance with Table 2. Cement paste without aggregate was mixed at a water to binder ratio of 1.25, and the size of the pastes utilized here was 20 × 20 × 20 mm, which was convenient to conducting these micro experiments, such as when using an X-ray diffractometer. The preparation and test of compressive strength were conducted in accordance with EN 196-1 [39], and the mortar size was 40 × 40 × 160 mm. In order to avoid further hydration, a small block that was smaller than 5 mm in the center of the same sample was immersed in anhydrous alcohol for 3 days prior to microanalysis. For further investigation, the residue samples were cured in slandered condition. It is important to remember that on the first day, the anhydrous alcohol needs to be replaced at 6 and 24 h, respectively. The materials were then dried for the test under vacuum conditions for more than 24 h at 45 °C to avoid the decomposition of hydrates.

### 2.4. Characterization Methods

#### 2.4.1. Powder Dissolution

**Figure 5:**
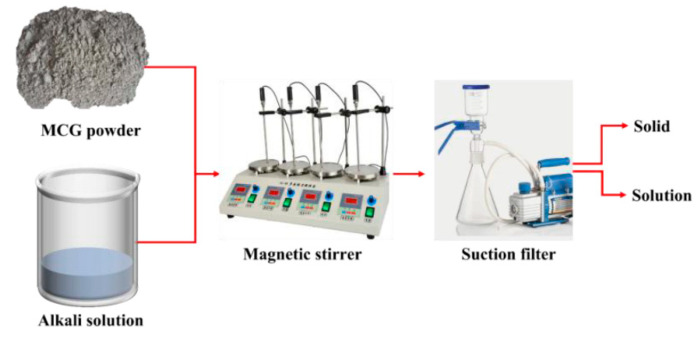
Flow chart of the dissolution process.

The flow chart of dissolution experiment, which is detailed in Figure 5, was performed to characterize CG activity. During the procedure, 100 mL of NaOH solution and 1 g of CG powder were prepared and swirled for a predetermined amount of time using a magnetic stirrer at various temperatures. The solid and solution phases were then separated using section filler, and the dissolved composition could be determined using the weight of the solid residue.

#### 2.4.2. Chemical and Mineral Compositions

The D8-Discover X-ray diffractometer (Bruker, Mannheim, Germany) was used for the XRD analysis, which used Cu-K radiation at 40 kV and 30 mA. The samples were initially mashed and sieved using a 200 mesh. The scanning range was 5–70°, the scanning speed was 0.15 s/step, and each step was 0.02° long.

Moreover, Nicolet iS10’s FT-IR test was used to analyze the components of MCG (Nicolet-iS10, Thermo Scientific, Waltham, MA, USA). Approximately 0.5 mg of the MCG sample was ground with 199.5 mg of potassium bromide (KBr) using an agate mortar and pestle to make the KBr pellet. Prior to each measurement, the background spectrum was scanned using the same instrumentation. The background spectrum was removed from the spectrum of each sample. In addition, all of the samples were scanned 32 times in the spectral region of 500–4000 cm^−1^ at a resolution of 4 cm^−1^.

#### 2.4.3. Particle Size Distribution

The Omec LS609 (Omec LS609, Spectris Inc, London, UK) apparatus was used to measure the particle size distribution of the PC and CG using the laser scattering technique. The samples were moved to the measurement cell after drying at 105 °C for 2 h. Three samples were examined.

#### 2.4.4. Microstructure and Morphology

To examine the surface morphology of materials, a scanning electron microscope (SEM) (FEI Nova Nano SEM 450, FEI, Hillsboro, OR, USA) with elemental mapping and EDX was employed. The secondary electron pictures were gathered at a 15 KV accelerating voltage. Prior to analysis, the CG or BCM samples were dried for 3 days in a vacuum drying oven, and then gold was sprayed over the sample surface using a sputtering coater (Q150T S, Quorum Technologies, Laughton, UK).

#### 2.4.5. Specific Surface Area

With Autosorb-IQ2 (Autosorb-IQ2, Quantachrome Instruments, Beach, FL, USA), a BET (Brunauer–Emmett–Teller) test was performed using N_2_ as the environment to determine whether the surface area and pore of the CG had changed after modification. N_2_ adsorption-desorption isotherms for all samples were measured at 77 K with relative pressures (P/P_0_) ranging from 0.05 to 0.995. Using the N_2_ adsorption data, the specific surface area was estimated using the multipoint BET technique.

#### 2.4.6. Thermodynamic Model Simulation

The mineral composition of the BCM with various reaction degrees was ascertained using the GEMS (http://gems.web.psi.ch/ (accessed on 22 May 2017)), Paul Scherrer Institute, Switzerland) [40,41]. GEMS is a Gibbs Energy Minimization program package for interactive thermodynamic modeling of heterogeneous aquatic (geo) chemistry systems, particularly those including solid solution-aqueous solution equilibria, adsorption/ion exchange, metastability, and dispersity of mineral phases.

In this experiment, the simple ideal mixing model was used in GEMS to represent the considered solid solutions of all the samples. During BCM hydration, the ambient temperature and pressure were 20 °C and 0.1 MPa (1 bar), respectively. Cemdata18 [42] was the database used in this investigation. Free downloads of the Cemdata18 database for GEM are available at http://www.empa.ch/cemdata (accessed on 8 January 2019), and it is completely compatible with the GEMS version.

The following are the calculated steps of this study: First, T the CG was defined firstly as raw material in GEMS according to Table 1. Then, all the components required to describe the hydration processes were chosen, including H, C, N, O, Na, Mg, Al, Si, S, Cl, K, Ca, and Fe. The activity and ionic strength of the ions, such as Ca^2+^, were calculated using the Extended Debye–Hückel model in Equation (2). Afterwards, the mix proportions for the pastes (Table 2) were next input into GEMS and the reactions were performed.

(2) $$\log\gamma =\frac{-AZ^{2}\sqrt{I}}{1+Ba\sqrt{I}}+bI$$

where *γ* is the activity coefficient, *A* and *B* are the parameters, *a* is an average distance of approach of two ions of opposite charge (or the ion-size Kielland’s parameter for individual ions), *b* is a semi-empirical parameter (~0.123 for KOH and ~0.098 for NaOH electrolyte at 25 °C), and *I* is the effective molal ionic strength.

#### 2.4.7. Compressive Strength

Using a universal testing device, the compressive strength test was performed from 3 to 28 days (WAW-1000D, Jinan, China). The findings were averaged after the loading rate of 2.5 mm/min was applied to the total of 3 compressive strengths. The compressive strength was calculated using the following Formula (3).

(3) $$\sigma =\frac{F}{\pi R^{2}}$$

where *F* is the peak load (N) and *R* is the radius of the cylindrical sample,

## 3. Results and Discussion

### 3.1. Dissolution Property of Chemically Modified CG

**Figure 6:**
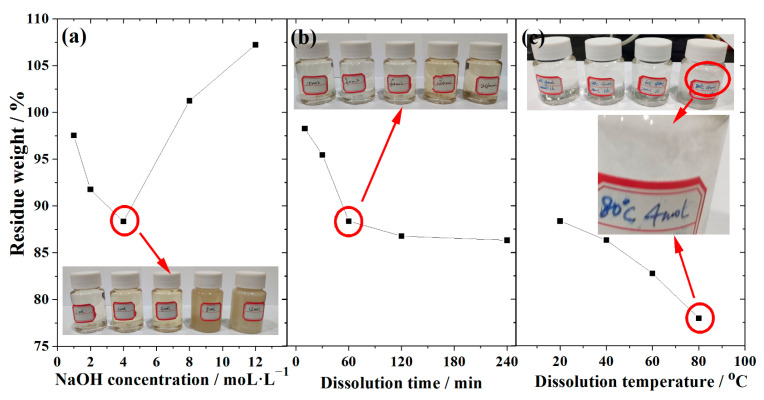
Dissolution property of CG at different (**a**) NaOH concentration, (**b**) dissolution time, and (**c**) temperature.

Dissolution method is widely applied in characterizing the activity of supplementary cementitious materials [43,44]. Comprehensive research is done into how the CG dissolves at various NaOH concentrations, temperatures, and times. The results are displayed in Figure 6. As shown in Figure 6a, the residue weight decreases with NaOH concentration until the NaOH concentration reaches 4 mol/L, from which point the residue weight changes slightly with NaOH concentration. The reasons are that: (1) at low NaOH concentration, the active components in CG dissolve into the NaOH solution without hydrates formation, which is the cause of the weight loss. (2) High NaOH concentration causes the concentration to oversaturate and the dissolving products/hydrates to precipitate on the CG, increasing the weight. These findings are supported by previous work [44,45].

The influence of dissolution duration on the CG dissolution property was examined in Figure 6b, where the dissolution temperature and NaOH concentration were fixed at 20 °C and 4 mol/L, respectively. It demonstrates that the residue weight decreases as the dissolving period increases until it reaches 60 min, after which point it slightly alters, demonstrating that the dissolution of CG in NaOH may be separated into two stages. (1) From mixing to 60 min, the active components in CG continue to dissolve into the NaOH solution at this stage, without the hydrates precipitating. (2) Slow dissolved stage, lasting 60 min or longer; from this time, the majority of the CG’s active ingredients have dissolved, and some hydrates have precipitated on the CG particles, impeding the remaining active ingredients’ ability to dissolve. Some earlier studies have also reported on comparable response models [13,45].

In Figure 6c, the temperature range of 20 to 80 °C with steps of 20 °C was examined. The NaOH concentration and dissolving time were fixed at 4 mol/L and 60 min, respectively. It demonstrates that the residual weight drops from 88.35% at 20 °C to 77.95% at 80 °C, demonstrating that temperature has a significant impact on the CG’s ability to dissolve. This is due to the Arrhenius Formula (4), which states that the activity of the elements or minerals is determined by *K* and *E_a_*, in which *E_a_* is only impacted a little by activation energy whereas *T* is heavily dependent.

(4) $$K=A\cdot e^{-\frac{E_{a}}{RT}}$$

where *K* is the reaction rate; *A* is a constant; *E_a_* is the activation energy; *R* is ideal gas constant; and *T* is the reaction temperature.

**Figure 7:**
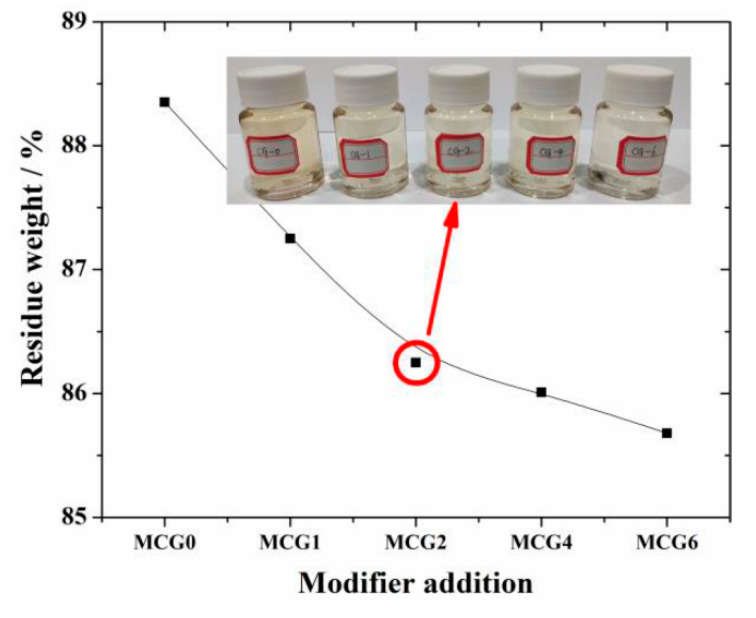
Dissolution property of MCG with different FC additions.

Based on the aforementioned findings, the dissolution characteristics of the MCG with various FC modifiers were examined under the following conditions: the temperature was set to 20 °C, the NaOH concentration was 4 mol/L, and the dissolution period was 60 min. The results are given in Figure 7. The results demonstrate that the residue weight decreases with the addition of FC, indicating the CG activity is improved after FC modification. The reason for this improvement may be that FC reacts with some minerals in CG and the active components are activated; this will be discussed in more detail in the following section. The drop in residue slows down when the modifier concentration rises over 2 wt.%, indicating that 2 wt.% is the ideal modifier addition to activate CG.

### 3.2. Composition and Morphology of MCG

#### 3.2.1. Composition of MCG

**Figure 8:**
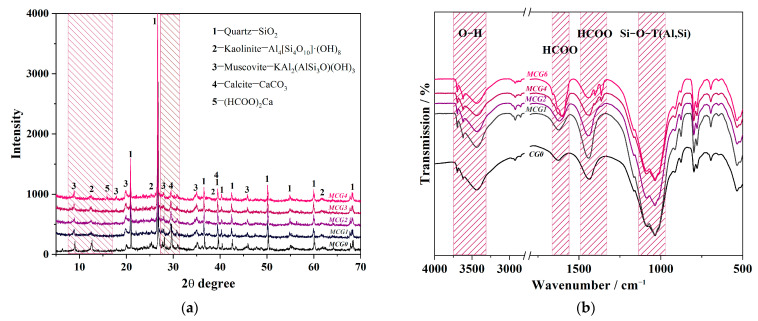
(**a**) XRD and (**b**) FTIR patterns of MCG with different FC additions.

The mineral composition and chemical bonding of MCG were evaluated by XRD and FTIR analysis, respectively, to determine the impact of FC on the composition of CG. The results are displayed in Figure 8a,b. According to the findings of Li et al. [45] and Xie et al. [13], CG is primarily composed of quartz, kaolinite, muscovite, and calcite, as shown in Figure 8a. The peak intensities of the minerals of kaolinite, muscovite, and calcite steadily decline as FC addition rises, meaning that the content of them has decreased. Moreover, a little peak associated with (HCOO)_2_Ca has emerged following FC modification, showing that the reactions between these minerals and FC as specified in Equations (5)–(7) have taken place. The active elements, such as Si, Al, and Ca, can dissolve more readily after modification, as seen by the decrease in residue weight in Figure 7.

Chemical reaction of kaolinite with FC:

Al_4_[Si_4_O_10_]·(OH)_2_ + HCOOH → (HCOO)_3_Al + H_10_[Si_4_O_10_] + H_2_O (5)

Chemical reaction of muscovite with FC:

KAl_2_(AlSi_3_O)(OH)_3_ + HCOOH → (HCOO)_3_Al + HCOOK + H_4_(AlSi_3_O) + H_2_O (6)

Chemical reaction of calcite with FC:

CaCO_3_ + HCOOH → (HCOO)_2_Ca + CO_2_ + H_2_O (7)

According to Figure 8b, the Si-O-T(Al,Si) between 1000 and 1200 cm^−1^, the O-H between 3300 and 3700 cm^−1^, and the HCOO between 1300 and 1800 cm^−1^ make up the majority of the FTIR patterns of CG [33,46]. Comparing the CG etched with different FC dosage, it shows that the HCOO peak that belonging to HCOOH, HCOOK, (HCOO)_3_Al, (HCOO)_2_Ca during 1300–1500 cm^−1^ and 1600–1800 cm^−1^ increases with the FC addition, while the OH peak that belongs to Al_4_[Si_4_O_10_]·(OH)_2_ and KAl_2_(AlSi_3_O)(OH)_3_ have decreased, indicating that the kaolinite, muscovite, and calcite have reacted with the FC. These outcomes match the XRD outcomes.

#### 3.2.2. Morphology, Particle Size, and Specific Surface Area of MCG

**Figure 9:**
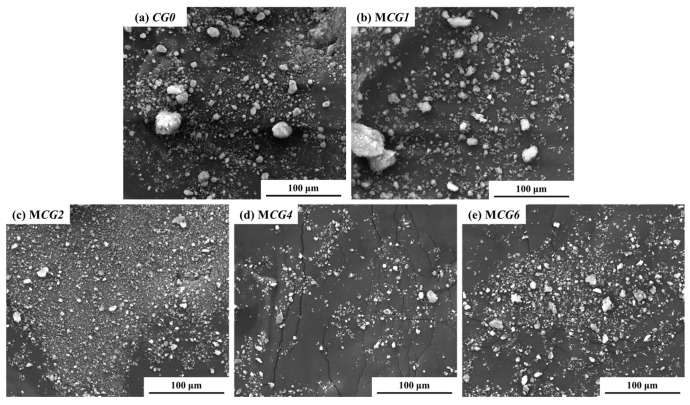
SEM of MCG with different modifier additions.

**Figure 10:**
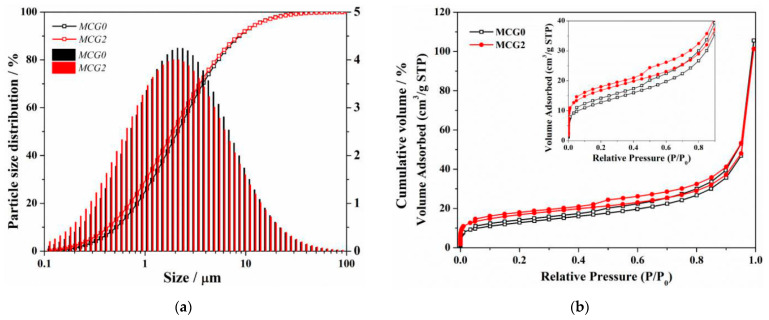
(**a**) Particle size distribution and (**b**) BET result of MCG.

SEM is used to describe the shape of the MCG in addition to its composition. At 1000× magnification, as seen in Figure 9, the inclusion of FC dramatically alters the CG’s shape. First off, MCG0 and MCG1 have substantial levels of large-scale particle content with maximal MCG particle diameters of 50 to 70 μm. The MCG particle surface is also uneven and rough. The maximum diameter of MCG2, MCG4, and MCG6 particles clearly reduces after being treated with 2 wt.%, 4 wt.%, and 6 wt.% FC, while the small-scale MCG content rises. The results are further supported by Figure 10a, where it is clear from the particle size distribution results that the changed procedure reduces the content of the particle size distribution, and the content of CG, in which particle sizes smaller than 10 μm increased.

Based on the results of the aforementioned experiment, it was determined that adding 2 wt.% of FC to the CG significantly improved its dissolving properties and morphology. As a result, the specific surface area of the MCG was further measured by the BET method, as shown in Figure 10b. The data in it shows that FC modification increases the CG absorbed capability at all pressures between 0.05 and 0.995 P/P_0_, demonstrating an increase in specific surface area. This can be explained in two ways: first, the alteration reduces the size of the CG particles, and second, certain in situ pores are created following the modification process. This has also been supported by other investigations [33].

### 3.3. Compressive Strength of BCM Prepared with MCG

**Figure 11:**
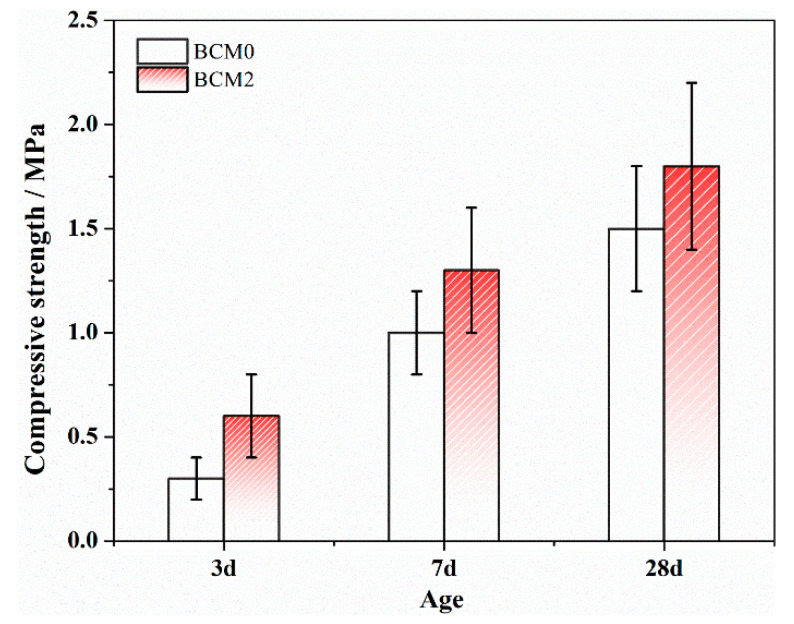
Compressive strength of BCM.

Ermolovich [47] recommend testing the performance of backfill materials up to 56 and 84 days, while in this investigation, the activity of the gangue is improved. As a result, early-age (from 3 to 28 days) performance should be paid more attention to. The key property of the materials used in the applications is compressive strength. From 3 to 28 days, the compressive strength of the BCM was thoroughly studied, with the results displayed in Figure 11. It demonstrates that BCM2 has a higher compressive strength than BCM0 at all ages; after three days, it reaches 0.6 MPa, or about two orders of magnitude more than the value before FC modification. This is due to the fact that modification chemicals such as calcium formate are likely to promote the hydration of tricalcium silicate after modification [48]. These effects will increase the content of hydrates produced during early hydration, improving the BCM’s compressive strength [49,50].

Although the 28 days compressive strength of the BCM2 is higher than the BCM0, the increased value is not noticeable in comparison to the samples at 3 days because the dense hydrate layer formed at an early age will prevent the inner minerals from hydrating, which is consistent with the findings in Ref. [51].

### 3.4. Hydrates and Microstructure of BCM Prepared with MCG

#### 3.4.1. Hydrates of BCM

**Figure 12:**
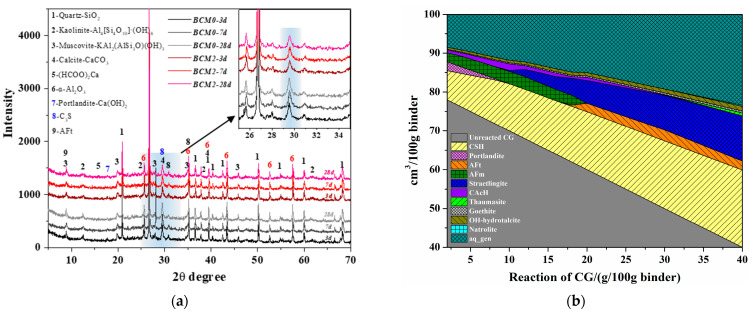
(**a**) XRD patterns of BCM at different ages and (**b**) mineral composition of BCM with CG reacted.

The hydrates of the BCM samples were analyzed by XRD in order to determine the hydrates evolution, and the findings are given in Figure 12a. The C_3_S and Ca(OH)_2_ peaks in BCM2 are found to be lower than BCM0 at all ages, indicating a decreased C_3_S and Ca(OH)_2_ concentration. According to Ref. [52], the C_3_S hydration results in C-S-H gels and Ca(OH)_2_, which bind various PC or CG particles together. The formate created during the modification process functions as an accelerator for hydration, which is the cause of the C_3_S belonging to PC hydrating more quickly than usual. Additionally, the results in Figure 8a,b are confirmed by the fact that BCM2 has lower kaolinite and calcite intensities than BCM0 at roughly 12.5° and 39.5°, respectively.

The amorphous C-S-H phases in the cement matrix could not be identified by XRD [53]. The GEMS was further conducted to investigate this modification and to quantify the minerals change in the hydration process. The result is depicted in Figure 12b. Because the dissolved Si and Al from the CG take part in the hydration given in Equations (8) and (9), it demonstrates that in 100 g of binder, the content of C–S–H gels increases with the reaction of CG. Ca(OH)_2_ disappears in this reaction after reacting with CG until it reaches around 10 g, as shown by the XRD patterns (Figure 12a).

C_3_S + H_2_O → C-S-H + Ca(OH)_2_ (8)

Amorphous phase + Ca(OH)_2_ → C-(A)-S-H + H_2_O (9)

#### 3.4.2. Microstructure of BCM

**Figure 13:**
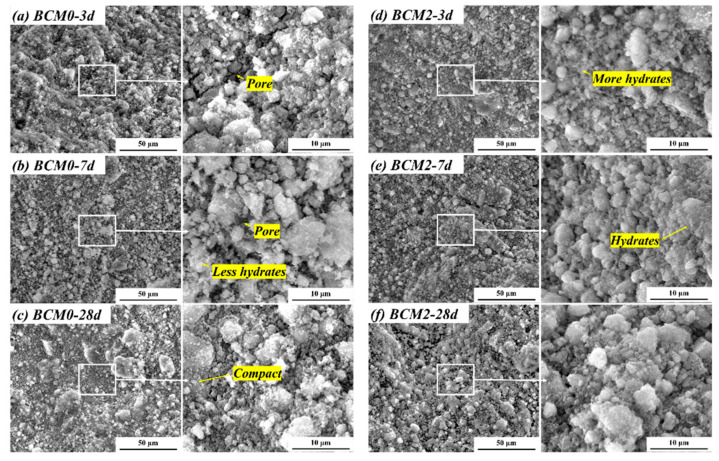
Microstructure of BCM.

The hydrates grow as they become more hydrated and subsequently fill the space left by the BCM. This process enhances the samples’ microstructure at the microscopic level and causes a rapid rise in compressive strength over the course of 3 to 28 days. Both the morphology of hydrates at matching ages and the microstructure of BCM were scanned and analyzed. As shown in Figure 13, the BCM0’s pores are clearly apparent at 3 and 7 days, and a few hydrates covering the PC or CG particles have been discovered. As a result, the BCM0’s structure is loose. While the connection between the various particles becomes tighter as a result of the CG being etched with FC, leading to more hydrates covering the PC or CG particles in BCM2 at 3 and 7 days. This results in reduced pore space in BCM2.

However, the difference in hydrates morphology and the microstructure in BCM0 and BCM2 at 28 days is not obvious. This is because the cement particles have hydrated to a high degree after 28 days, resulting in the nearly identical hydrates content in the two samples. As a result, the SEM images show that the microstructure difference between BCM0 and BCM2 is not visible. The results of compressive strength indirectly reflect these microstructure variances (Figure 11).

### 3.5. Modification and Hydration Mechanisms for MCG

**Figure 14:**
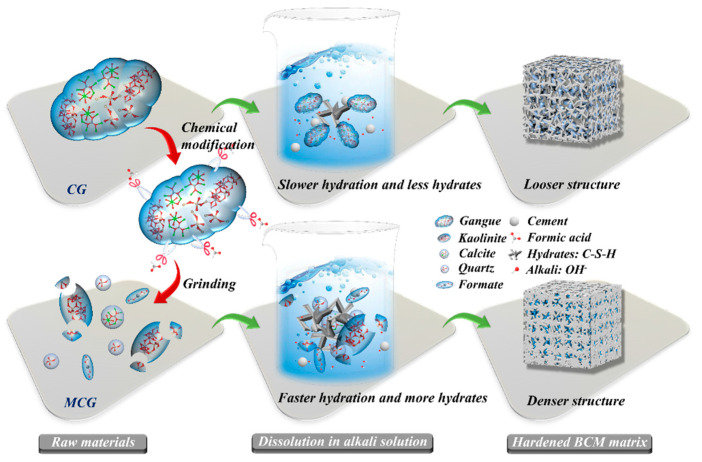
Schematic diagram of modification CG and hydration of BCM.

The procedures for chemical modification, MCG dissolution, and BCM hydration are methodically described in Figure 14. It indicates that a CG particle is composed of a variety of minerals, including kaolinite, calcite, and quartz, which are known to be the primary constituents of CG in earlier studies [54,55]. The fact that these different minerals tend to aggregate together causes the CG particle size to be quite large and the CG activity to be very low, as demonstrated in numerous literature sources [22,24]. Yet, when employing FC to modify the CG, the FC functions like a pair of scissors during the modification process, slicing up the CG and using grinding to disassemble the kaolinite and calcite’s structure, reducing the CG’s particle size. In addition, because of how the minerals react during the modification process, in situ pores will show up. The BET result and our prior study [33] have both confirmed that these two impacts will enhance the specific surface area of the CG. In addition, the FC reacts with calcite or kalinite to produce formate, which is a byproduct that is comparable to calcium formate, and these byproducts will be essential in the subsequent hydration process, which can aid in cement hydration.

According to Li et al. [43] and Irannajad et al. [56], unmodified CG has a low degree of reactivity, which makes it difficult for the active components to dissolve and conduct reactions. In the dissolution and hydration processes, the cement and CG particles dissolve in water to create C-S-H and Ca(OH)_2_, which raises the pH of the solution. The formation of the microstructure and compressive strength is primarily influenced by the amounts of C-S-H and Ca(OH)_2_. Si and Al elements in MCG particles are easier to disperse into the solution than CG is in such an alkaline solution due to the FC modification effect, which shrinks the CG particle size and destroys the mineral structure.

Cacciuttolo et al. [57] noted that the compressive strength of backfill pastes is critically important for the BCM application, which will decide its use in controlling strata movement. Since it is known that the quantity of C-S-H/C-(A)-S-H increases with the cement [58], these hydrates act as links to connect different particles, fill the pores of the BCM, and help the BCM gain strength. BCM0 has more pores and fewer hydrates than BCM2, which is due to the poor activity of CG. BCM2’s microstructure has significantly improved after being etched with FC, and as a result, BCM2’s compressive strength is higher than that of BCM0.

## 4. Conclusions

In this study, a novel approach, chemical modification, was applied to activate the gangue, and chemically modified CG was used as a basic material for the production of cement backfill paste. To better understand the properties of MCG, the dissolution property of MCG and hydration characteristics of MCG-based backfill pastes were comprehensively investigated in this paper. The takeaways from the study are as follow:

(1) The dissolution of CG is obviously influenced by the NaOH content, dissolving duration, and temperature. The dissolution property of CG can be further enhanced by dry chemical modification. The residual of CG reaches a satisfactory value at 2 wt.% FC based on CG.
(2) The CG’s structure is destroyed when the FC reacts with the calcite and kaolinite to produce formate, which allows the CG’s particle size to be further reduced when combined with grinding and also increases the CG’s specific surface area.
(3) At 3, 7, and 28 days, BCM prepared with MCG has a compressive strength that is higher than that of the control group. The micro studies demonstrate that the formate accelerated action causes a decrease in C_3_S and Ca(OH)_2_ content in BCM, resulting in an increase in C-S-H gels and a denser microstructure of BCM.
(4) In conclusion, dry chemical modification is a promising strategy that might enhance the activity of CG and enhance the microstructure and compressive strength of BCM made with MCG.

The study also has several limitations. First, this present study only focused on the early-age performance of BCM, and did not pay more attention to the long-term performance of BCM. Second, the impact of BCM on Sustainable Development Goals (SDGs) should be discussed further.

Despite these limitations, the study has important implications to theory and practice. First, the study enriches the theory of how to improve CG’s reactivity. Second, this work provides a unique method of activating CG, and then makes a comprehensive study on the compressive strength and microstructure of BCM made from activated CG. 

## Figures and Tables

**Table 1:** Chemical compositions of PC and CG, wt.%.

Oxide	CaO	SiO_2_	Al_2_O_3_	Fe_2_O_3_	MgO	Na_2_O	K_2_O	SO_3_	LOI	Total
PC	64.65	21.88	4.49	3.45	2.36	0.51	/	2.44	1.31	100
CG	2.76	65.06	24.07	4.22	0.71	0.62	2.06	0.50		100

**Table 2:** Mix proportion of BCM pastes (without aggregate) and mortar, g/100 g.

Serials	PC	Modified CG	Modified CG Addition	1–2 mm CG Aggregate	Water
BCM0	3.2	CG0	12.8	64	20
BCM1	3.2	MCG1	12.8	64	20
BCM2	3.2	MCG2	12.8	64	20
BCM4	3.2	MCG4	12.8	64	20
BCM6	3.2	MCG6	12.8	64	20

## Data Availability

Data are contained within the article.
